# Characterization of the octamer, a *cis*-regulatory element that modulates excretory cell gene-expression in *Caenorhabditis elegans*

**DOI:** 10.1186/1471-2199-11-19

**Published:** 2010-03-08

**Authors:** Allan K Mah, Domena K Tu, Robert C Johnsen, Jeffrey S Chu, Nansheng Chen, David L Baillie

**Affiliations:** 1 Department Molecular Biology and Biochemistry, Simon Fraser University, 8888 University Drive, Burnaby, British Columbia, Canada, V5A 1S6; 2 Department of Medical Genetics, Centre for Molecular Medicine and Therapeutics, University of British Columbia, 950 West 28th Avenue, Vancouver, British Columbia, Canada V5Z H4H

## Abstract

**Background:**

We have previously demonstrated that the POU transcription factor CEH-6 is required for driving *aqp-8 *expression in the *C. elegans *excretory (canal) cell, an osmotic regulatory organ that is functionally analogous to the kidney. This transcriptional regulation occurs through a CEH-6 binding to a *cis*-regulatory element called the octamer (ATTTGCAT), which is located in the *aqp-8 *promoter.

**Results:**

Here, we further characterize octamer driven transcription in *C. elegans*. First, we analyzed the positional requirements of the octamer. To do so, we assayed the effects on excretory cell expression by placing the octamer within the well-characterized promoter of *vit-2*. Second, using phylogenetic footprinting between three *Caenorhabditis *species, we identified a set of 165 genes that contain conserved upstream octamers in their promoters. Third, we used promoter::GFP fusions to examine the expression patterns of 107 of the 165 genes. This analysis demonstrated that conservation of octamers in promoters increases the likelihood that the gene is expressed in the excretory cell. Furthermore, we found that the sequences flanking the octamers may have functional importance. Finally, we altered the octamer using site-directed mutagenesis. Thus, we demonstrated that some nucleotide substitutions within the octamer do not affect the expression pattern of nearby genes, but change their overall expression was changed. Therefore, we have expanded the core octamer to include flanking regions and variants of the motif.

**Conclusions:**

Taken together, we have demonstrated that octamer-containing regions are associated with excretory cell expression of several genes that have putative roles in osmoregulation. Moreover, our analysis of the octamer sequence and its sequence variants could aid in the identification of additional genes that are expressed in the excretory cell and that may also be regulated by CEH-6.

## Background

The *Caenorhabditis elegans *excretory system is composed of four cells: the excretory duct cell, the bi-nucleate excretory gland cell, the excretory pore cell, and the excretory (canal) cell (EC). Each of these cells are descendents of the AB cell lineage [1]. The EC in particular has a unique H-shaped structure consisting of two pairs of bilaterally-symmetrical projections that protrude anteriorly and posteriorly from the central cell body. The EC forms approximately 270 minutes after the first cellular division near the centre of the embryo [2]. Subsequently, two processes extend dorso-laterally, which then bifurcate to form the anterior and posterior canal branches. By the end of the first larval stage, the EC canals have reached their full length relative to the length of the nematode [3]. Further growth of the canals is influenced by their attachment to the hypodermis, which promotes extension of the EC canals following the first larval stage [3]. Not surprisingly, because of similarities in their structures, the EC and neurons share many developmental cues that dictate elongation, guidance and outgrowth [4].

Even though the EC shares developmental cues with neurons, it has a distinct function. The EC plays a role in maintaining osmotic balance by collecting soluble organic and inorganic metabolic waste substances and expelling these to the environment [5]. For *C. elegans*, osmoregulation is a critical function due to the continuous and unpredictable stresses placed upon worms in their native soil habitat. To facilitate the exchange of dissolved material, the fluid-filled EC arms are exposed to pseudocoelomic fluid. The pseudocoelomic fluid in turn is in contact with most of the cells in *C. elegans*, presumably having a function analogous with circulatory fluid. The pseudocoelomic fluid also provides the turgor for the hydrostatic skeleton of *C. elegans*, which is essential for locomotion. In addition to maintaining osmotic balance, the excretory system is responsible for secreting hormones [6] and fluids required for molting [7]. Notably, the osmoregulatory function of the EC resembles the role of the mammalian kidneys. Thus, characterizing conserved mechanisms that govern EC transcription may provide insight into regulatory circuits that control kidney-specific transcription.

We are interested in the mechanisms that govern transcriptional regulation in the EC of *C. elegans*. Our laboratory has previously characterized two distinct transcriptional regulatory mechanisms affecting EC gene expression. One of these mechanisms relies upon binding of the transcriptional regulator DCP-66 to the Ex-1 *cis-*regulatory element (CCATACATTA). Together, DCP-66 and Ex-1 drive EC-exclusive expression of *pgp-12*, an ABC transporter-encoding gene [8]. However, DCP-66 is a component of the transcriptional inhibitory nucleosomal remodeling and deacetylase (NuRD) complex, which is typically associated with gene repression [9]. The second mechanism, involves CEH-6, a class III POU homeobox transcription factor (TF), which binds to a *cis-*regulatory element called the octamer (ATTTGCAT) [10]. We originally characterized the octamer as an element required for EC-expression of the aquaporin-encoding gene *aqp-8*. Of note, CEH-6 is also expressed in the excretory cell (EC), thus fulfilling the spatial requirements of a TF responsible for EC-selective transcription. Additionally, CEH-6 is detected in the bilaterally symmetric neurons (RMDDLR, RMDVLR, AUALR and AVHLR), P.na cells (ventral nerve cord), five rectal cells (B, Y, U, F and K) [11], two tail nerve cells, ventral hypoderm, anterior body wall muscle, body wall muscle cells, and intestine [12]. The broad expression of CEH-6 indicates that it likely regulates transcription in a several ectodermal cells as well.

Because CEH-6 interacts with the octamer to drive *aqp-8 *expression in the EC, we wanted to determine whether the octamer is generally linked to EC-expression. If so, octamer regulated genes could represent novel candidates that function in the EC. Furthermore, we also attempted to define the role of the octamer *cis*-regulatory element as a driver of EC-selective transcription. Specifically, we determined whether the octamer is under spatial restrictions within promoter regions, identified genes that require the octamer for EC expression, and identified variants of the octamer that are able to drive EC-expression. Thus, this work identified a set of candidate genes that could be relevant to kidney function in vertebrates. Overall, our data reinforce the role of octamers and presumably their cognate transcription factors such as CEH-6 in directing osmoregulatory organ gene expression.

## Results

### The Location of the Octamer Sequence in the Promoter can be Flexible

**Figure 1:**
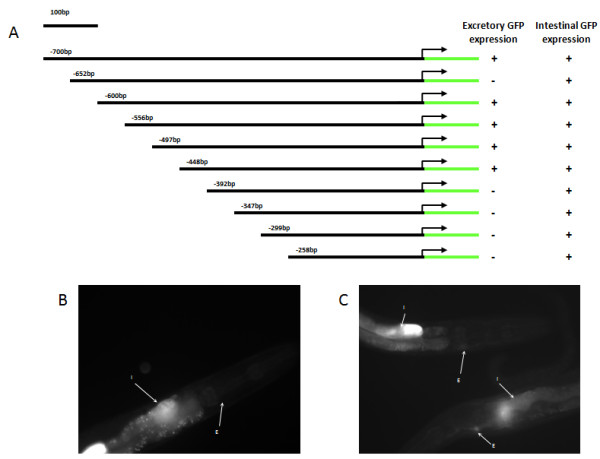
**Effects of the octamer at various distances upstream of a gene's translational start site**. We placed the octamer upstream of various lengths of *vit-2 *promoter fragment to assay the ability of the octamer to drive EC-expression at different places within promoters. *A*, Octamers were appended onto the 5' end of decreasing *vit-2 *promoter-regions. The number represents the 5'-position of the *vit-2 *promoter fragment (distance upstream indicated). *B*, All *vit-2 *constructs less than and including the -392 bp (shown) construct failed to result in EC-expression although each of these constructs still have reporter expression in the intestine (I). *C*, Constructs with *vit-2 *upstream regions larger than and including the -448 bp (shown)construct had GFP expression in the EC (E) in addition to the expected expression in the intestine. The exception was the *vit-2 *promoter construct with the -652 bp 5'-end which failed to drive EC expression. * Both fluorescent images (*B *and *C*) were captured using 2 second exposure times.

Previously, we found evidence that the octamer is spatially restricted in the *aqp-8 *promoter [10]. However, we also determined that the octamer still had the capacity to drive EC-specific expression when placed in close proximity to the ATG using the *Δpes-10 *minimal promoter [10] (note that we refer to the ATG because many transcriptional start sites are poorly characterized in *C. elegans*). Thus, we hypothesized that the octamer might have different spatial restrictions in the promoter regions of different genes. To assess spatial dependence of the octamer within the promoter region, we used the *vit-2 *promoter as a tool. *vit-2 *encodes a yolk protein that is expressed at high levels in the intestine [13], but is not normally expressed in the EC. A promoter encompassing 247 bps directly upstream of the *vit-2 *ATG is sufficient for driving intestinal expression of *vit-2 *[14]. Therefore, we used the intestinal expression to assay for promoter function. We generated a series of promoter constructs by appending tandem octamer sequences to the 5' end of *vit-2 *promoter truncation constructs (258 bp to 700 bp upstream of the ATG) (Figure 1A). These constructs were fused to GFP and then injected into wild-type worms to generate transgenic nematodes. Subsequently, we monitored for GFP expression in the intestine and in the EC.

We observed that the octamer was not able to drive EC expression in constructs that contained less than 448 bp upstream of the *vit-2 *ATG. However, these constructs retained the ability to drive intestinal expression indicating that the transgene was successfully generated and functional (Figure 1B). Placing the octamer at the 5'-end of *vit-2 *promoter constructs larger than 448 bp upstream of the ATG led to ectopic GFP expression in the EC (Figure 1C). An exception to this was from a transgene containing octamers 652 bp upstream of *vit-2*'s ATG, which did not drive assayable levels of GFP. This transgenic strain drove intestinal GFP, but failed to drive GFP in the EC. The lack of EC-expression indicates that there may be a *cis*-linked element located between 600 bp and 652 bp upstream of the *vit-2 *ATG that represses EC expression. Alternatively, it is possible that this transgene construct formed a concatemer *in vivo *that is incompatible with the expression of the GFP reporter. This second scenario is unlikely because we did observe intestinal expression. In any case, these data suggest that octamer function may be spatially restricted in some promoters. Taken together, although the above data did not allow us to conclude whether the octamer has an optimal distance from the ATG for influencing EC-expression, the data do suggest that functional octamers can be present at various places in different promoters.

### Many EC-expressed Genes are not regulated by Octamers

Because functional octamers may be located at various distances upstream of the ATG, we searched for genes with upstream octamers within 1,200 bp upstream of their ATGs; WormBase (WS195). Thus, we identified 1,340 candidate genes including promoters that contain either the forward and/or the inverse of the octamer (ATGCAAAT). To assess the function of these octamers, we selected genes from this set that are expressed in the EC [14,15]. In addition, we assessed the function of the octamer in the promoters of *hlh-8 *and ZC395.10, genes with no known EC-expression. For these seventeen genes, we tested if regions containing the octamer are required for EC-expression by truncating the promoter from the 5'-end and observing whether the octamer regions affect the level of EC expression (Additional file 1). However, we could not conclude whether the octamers were functional in promoters of Y69E1A.6, F36H1.2, Y48A6B.8, F29F11.6, B0334.4, C02B8.6, H23N18.3, R13F6.3, and, Y53G8AR.3 because EC expression was lost in worms carrying promoter constructs that still had the octamer, suggesting that there are other *cis-*linked elements that drive EC-expression of these genes (Additional file 1). Amongst the other eight genes, our promoter truncation analysis revealed three genes that are likely dependent upon the octamer containing region for EC expression: ZC395.10, C01B12.3, and *hlh-8*/C02B8.4. These results are not surprising as previous reports indicate that 12% of EC-expressed genes are predicted to be regulated by the DCP-66/Ex-1 mechanism [8] Additionally, the nuclear hormone receptor NHR-31 regulates most of the vacuolar ATPase (vATPase) components in the EC [16] pointing to multiple regulatory mechanisms that specify EC-expression.

**Figure 2:**
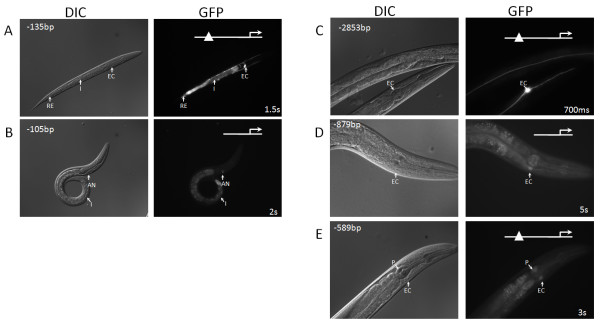
**Analysis of excretory cell expression-dependence on upstream octamers**. We identified several genes that require the octamer for proper levels of EC-expression. *A*, A -135 bp 5'-truncation of *ZC395.10*'s upstream region drives expression in the EC (EC) along with the rectal epithelia (RE) and intestine (I). *B*, A -105 bp 5'-truncation of *ZC395.10*'s region can still drive expression in the intestine, but EC and rectal epithelial expression is lost. *C*, A -2,853 bp 5'-end *C01B12.3 *drives relatively strong EC expression. *D*, Truncating the *C01B12.3 *promoter to 879 bp upstream of the ATG leads to a drop in the EC expression level. *E*. A -589 bp 5'-truncation of *C02B8.4*'s upstream region drives expression in the EC and the pharynx (P). Exposure times are indicated on the images.

The octamer in *ZC395.10*'s promoter region is located 120 bp upstream of the ATG. *ZC395.10 *is localized to most neurons, intestine, pharynx, and vulva [14,15]. However, a *ZC395.10 *promoter construct containing the octamer (5'-end 135 bp upstream of the ATG and in the forward orientation) drove expression in a different pattern than the original longer promoter. In this shorter promoter construct, we observed GFP in the EC, anterior neurons, intestine, and rectal epithelia (Figure 2A). The differences between this and the construct with the larger promoter indicate that there is a *cis-*linked repressor element(s) that modulates EC and rectal epithelial tissue expression in ZC395.10's promoter. Interestingly, the EC and rectal epithelia expression pattern resulting from the shorter construct overlaps with CEH-6's expression pattern [3]. Furthermore, deleting the octamer containing region leads to loss of expression in both the EC and rectal epithelia. Additionally, there must be an independent transcriptional regulatory mechanism that drives ZC395.10 expression in anterior neurons and the intestine (Figure 2B).

An octamer is located 1,055 bp upstream of the ATG in C01B12.3's promoter region. A transcriptional reporter construct with a 5'-end 2,853 bp upstream of the ATG leads to expression in the EC (Figure 2C), hypoderm, spermatheca, and the anal depressor muscle [14,15]. However, the EC-expression level is greatly decreased when the promoter is truncated (879 bp upstream of the ATG) corresponding to removal of a large portion of the promoter and including an octamer (Figure 2D). Interestingly, the orthologs of C01B12.3 in *C. briggsae *and *C. remanei *also have octamers in their promoters. Moreover, the distance of the octamers from the ATG are similar (1,108 bp and 1,100 bp upstream of the ATG respectively), suggesting that this element might also regulate EC-expression of C01B12.3 orthologs.

The *hlh-8 *promoter contains an octamer located 582 bp upstream of the ATG. It was previously shown that *hlh-8 *is expressed in the intestine, anterior neurons, and vulva [14,15]. A 5' truncation that limits the promoter to only seven bases upstream of the octamer (589 bp upstream of the ATG) results in expression localized to the EC and the second pharyngeal bulb (Figure 2E). This change in expression pattern indicates the likely presence of a repressor element(s) that blocks EC and pharyngeal expression. Expression of *hlh-8 *in the EC is completely abolished upon deletion of the octamer-containing fragment. In sum, our promoter deletion analysis identified octamer-containing promoter regions required for the EC-specific expression for the above three genes.

### Conserved Octamer sequences in Promoter Regions Strongly Bias for EC Expression

Due to the low success rate in finding octamer containing regions associated with EC-expression with the above approach (only 3/17), we turned to phylogenetic footprinting. Using this comparative method, we identified promoters with perfectly conserved octamers between three closely related *Caenorhabditis *species (*C. elegans*, *C. briggsae*, and *C. remanei*). This resulted in the identification of 165 promoters that contain conserved octamers (Additional file 2).

Of the 165 genes, nineteen genes had previously characterized expression patterns [14,15]. To obtain a larger sample size, we analyzed the expression patterns of an additional 88 candidates (Additional file 3). From these 107 promoters, we identified 64 that could drive detectable levels of GFP expression, including 25 (39%) that were EC-expressed. This represents a significant enrichment of genes expressed in the EC when compared to a control dataset of 1,885 expression patterns, within which only 10.2% (193/1,885) of all genes are expressed in the EC [14,15]. Strikingly, twelve genes (19%) in our set were expressed only in the EC. This is a vast enrichment over the control set which contains only 0.3% (6/1,885) genes with EC-exclusive expression [14,15] (significance P < 0.01 as determined by 1-tailed Z-test).

**Figure 3:**
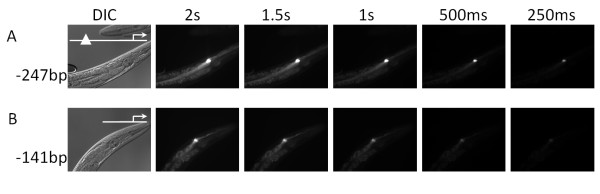
**The level of EC expression is decreased upon loss of the octamer in the region upstream of *C05D12.1***. The octamer is located 205 bp upstream of the ATG of *C05D12.1*. Loss of the octamer leads to a decrease in the GFP expression level. *A*, A -247 bp 5' truncation leads strong expression localized to the EC. *B*, A -141 bp 5' truncation leads to a lower level of expression, but still localized to the EC.

Next we wanted to determine whether their EC-expression was dependent on the octamer containing fragments. Using the same approach as in the previous section (5' serial promoter truncations), we selected 21 promoters that drove EC expression for further analysis (Additional file 4). From this set, we identified four genes that are completely dependent on fragments containing the octamer for EC expression (M176.*5*, *aqp-8*/K02G10.7, *twk-36*/R12G8.2), C05D12.1) and two genes that exhibit reduced EC expression upon deletion of the octamer region (R02F2.8, and F16F9.1) (Additional file 4; Figure 3) (note: the *aqp-8 *promoter was identified in the phylogenetic analysis, but was not subject to truncation analysis as it's octamer has been previously characterized [10]).

### Sequences Flanking Functional Octamers are Likely Conserved

**Figure 4:**
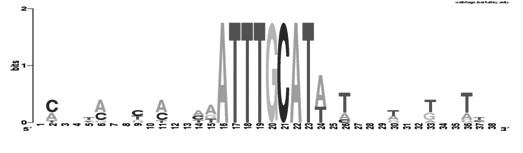
**Alignment of octamer and flanking regions of octamers responsible for EC expression reveals that flanking residues are A-T rich**. 15 bp upstream and 15 bp downstream flanking regions of the functional octamers were used for the WebLogo alignment http://weblogo.berkeley.edu/.

In the experiments described above, we identified genes that may depend on the octamer for expression in the EC. Thus, we used these promoters to study whether the sequences flanking the octamer are conserved. We aligned the nine octamer sequences along with 15 bp of upstream and downstream flanking sequences. The resulting alignments were displayed using WebLogo [17] (Figure 4; note that the reverse complementary sequence was used if the octamer was inverted). This analysis revealed that, in general, octamers are flanked by AT-rich regions. More specifically, regions upstream of the octamer are biased towards being A/C-rich and downstream regions tend to be T-rich. Thus, our newly identified collection of EC-expressed genes has allowed us to define additional specificity determinants related to the octamer.

### Intra-octamer Nucleotide Substitutions Have Different Effects on EC Expression

**Figure 5:**
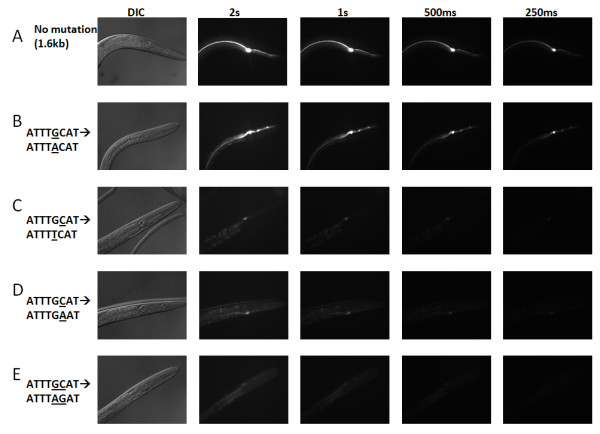
**Effects of octamer mutagenesis on expression levels**. We performed substitutions of residues within the octamer in *aqp-8*'s promoter region and assayed for changes in EC-expression. We targeted the -264G and -263C residues. The expression patterns were either not affected, diminished, or completely lost. *A*, *aqp-8*^*promoter*^*::GFP *reference strain. *B*, a -264G→A change led to no change in expression level. *C*, *D*, *E*, Mutations in the form of -264G→T, -263C→A, and -264GC→AG all lead to appreciable loss of GFP expression.

Previously, we observed that the octamer is perfectly conserved within the promoters of *aqp-8 *orthologs among five *Caenorhabditis *species [10]. Therefore, we hypothesized that the octamer sequence must be absolutely conserved to drive EC-specific expression. To examine the octamer in more detail, we manipulated its sequence using site-directed mutagenesis. We generated variants of the octamer by targeting nucleotides -264 bp and -263 bp of the octamer in the *aqp-8 *promoter (bold residues ATTT**GC**AT). Every possible single-nucleotide substitution and a dual nucleotide substitution were tested at these sites. The mutated constructs were fused to *GFP *to visualize changes in EC-expression. The 5'-end of every construct was defined as 276 bp upstream of the *aqp-8*'s ATG because truncation constructs ranging between 1.6 kb to 272 bp upstream of *aqp-8*'s ATG provide consistent EC-specific expression patterns [10]. Also, because the *aqp-8 *promoter produces consistent levels of EC expression, our reference transgene construct is a 1.6 kb *aqp-8 *promoter region fused to GFP (Figure 5A) [10].

At -264 bp, a G→A residue change (*aqp-8*^*promoter*(-264*G*→*A*)^*::GFP*) did not alter the expression level or pattern (Figure 5B). However, a G→T change (*aqp-8*^*promoter*(-264*G*→*T*)^*::GFP *led to a significant decrease in the EC expression level (Figure 5C). A G→C substitution (*aqp-8*^*promoter*(-264*G*→*C*)^*::GFP*) led to a complete loss of EC-expression. At -263 bp, a C→A residue substitution in *aqp-8*^*promoter*(-263*C*→*A*)^*::GFP *led to decreased EC-expression (Figure 5D). The C→G and C→T substitutions (*aqp-8*^*promoter*(-263*C*→*G*)^*::GFP *and *aqp-8*^*promoter*(-263*C*→*T*)^*::GFP*) both led to a complete losses of GFP expression. Finally, replacing the GC pair at -264 with an AG pair (*aqp-8*^*promoter*(-264*GC*→*AG*)^*::GFP*) led to decreased EC-expression (Figure 5E). In all constructs, the GFP signal remained localized to the EC suggesting that some octamer sequence variants have the capacity to influence EC expression.

## Discussion

In this study, we demonstrate that octamer containing regions are involved in driving EC-expression of several genes. This builds upon our previous data demonstrating that *aqp-8 *is dependent upon the POU homeobox TF CEH-6 and the octamer for EC-expression [10]. In several nematodes, the position of the octamer relative to the ATG is fairly well conserved among *aqp-8 *orthologs [10]. This is interesting as some *cis-*regulatory elements are spatially restricted within promoters. For example, in *C. elegans *functional X-box motifs cluster roughly 100 bp upstream of ATGs to drive neuronal expression [18]. Likewise, the GC-box *cis-*regulatory element has an optimal distance in relation to the TATA box. Moving the GC-box away from its optimal distance leads to decreased expression levels of nearby genes even though its cognate TF, Sp1, still bind with similar affinities [19]. In addition to spatial restriction relative to ATGs, relative positioning between *cis-*regulatory elements within the same promoter can affect expression. For instance, the β-actin promoter contains the so-called CCAAT and CCArGG boxes. These regulatory elements are binding sites for the TFs nuclear factor Y (NF-Y) and serum response factor (SRF), respectively. Manipulating the intra-element distance between these two *cis-*regulatory sequences accordingly affects β-actin message levels [20]. However, in the present study we had also observed that the octamer could be placed at different positions in a heterologous promoter and still drive EC-specific expression. Therefore, unlike the above examples, the octamer does not appear to be as spatially restricted within promoter although there still might be some tight limitations to octamer location.

In this study, we used different strategies to identify novel genes that require upstream octamer containing regions for expression in the EC. First, we identified genes with octamer sequences in their promoters. Using this strategy we identified a small number of genes that are modulated by upstream octamer-containing fragments. Secondly, we used a more stringent approach to identify octamers by relying on interspecies conservation. We discovered that the expression patterns of genes in this filtered promoter set had a higher than expected incidence of EC-expression. In these two screens, we identified nine genes that are likely octamer-modulated. Several of these genes likely have osmoregulatory functions, agreeing with the notion the EC is analogous to the kidney. In general, the genes we identified fell into four categories: transmembrane channels/pores, Hsp90 co-chaperones, proteins with unknown functions, and TFs.

The largest group of genes encodes transmembrane channels/pores, indicating that many genes regulated by the octamer participate in substrate transport. The five channels/pores are:

1) *twk-36 *encodes a *C. elegans *TWIK potassium ion channel protein. In vertebrates, TWIKs are commonly expressed in neuronal tissues and, to a lesser extent in lungs, skeletal muscle [21], and tubular portions of the kidney (proximal tubule, ascending limbs, distal convoluted tubules, and medullary collecting duct) [22]. The mammalian TWIK, TASK, is sensitive to changes in extracellular pH, indicating that some of these proteins have roles in modulating cellular responses to pH flux [23]. Also, because their conductance is osmotically regulated, TWIKs can influence cellular volume [24]. The *C. elegans *genome has 42 *twk *genes. As in other organisms, most of the *C. elegans twks *are expressed in neurons [25]; however, *twk-36 *is the only *twk *expressed in the EC [25]. Interestingly, another group has demonstrated that *twk-36 *is directly regulated by CEH-6 [26]. This independent study strengthens the notion CEH-6 is a *bona fide *regulator of EC expression that likely acts through the octamer in the promoter of *twk-36*. Therefore, it is possible that CEH-6 regulation impacts at least some of the octamer-dependent candidates identified here.

2) *aqp-8*, an aquaporin whose function and regulation have been characterized previously [10,27].

3) C05D12.1 is a homolog of the cytochrome b561/ferric reductase SDR-2. In mammals, SDR-2 is expressed in the brain [28] and kidney where it aids in iron reabsorption via the accessory transporter, divalent-cation transporter 1 (DCT-1) [29]. Cytochrome b561 proteins transport electrons in an ascorbate-dependent manner. Due to the role of SDR-2 in ascorbate regeneration, C05D12.1 could be involved in vitamin C homeostasis and/or oxidative stress responses.

4) R02F2.8 encodes a solute carrier (SLC) protein that is most similar to the mammalian SLC36 subfamily. SLC36 proteins are localized to intracellular and plasma membranes [30] where they function as symporters. SLC36 proteins transport small neutral amino acids such as glycine, alanine, and proline. Because SLC36 proteins affect proton flux, they also contribute to intracellular pH homeostasis [30]. In mammals there are four SLC36 genes, two of which are expressed in the kidney (SLC36A1 and SLC36A2) [31].

5) C01B12.3 encodes a *C. elegans *Bestrophin 3 homolog. Bestrophins are transmembrane proteins that modulate calcium dependent transport of chloride ions across cellular membranes. Bestrophins are enriched in the plasma membranes of epithelial cells where they manage cellular volume [32]. Bestrophins are also expressed in exocrine gland tissues (*e.g*. pancreas, lacrimal and salivary glands), lung, testis and kidney [33]. In these tissues they facilitate trans-epithelial movement of chloride ions leading to water and electrolyte movement [33].

In addition to transmembrane channels and pores, we uncovered several other genes that have less obvious links to osmoregulation and kidney biology. One of these, ZC395.10, is homologous to thehighly conserved Hsp90 co-chaperone protein, p23. p23 interactions with Hsp90 to ensure the proper folding and maturation of many proteins including steroid receptors [34], telomerase [35], and proteins that are upregulated in cancers [36]. We also identified M176.5, a gene with little prior functional data. M176.5 is a nematode-specific gene that is mainly composed of hydrophobic amino acids and is therefore likely to be localized to cell membranes and/or forms a globular protein.

Finally, we identified two TFs. The first, F16F9.1 is a homolog of the mammalian protein lipopolysaccharide-induced tumor necrosis factor-alpha factor (LITAF; a.k.a. SIMPLE/Small Integral Membrane Protein of Lysosome/Late Endosome) [37]. LITAF is linked to Charcot-Marie-Tooth (CMT1C) disease, a heritable neuropathy characterized by loss of muscle tissue and touch sensation [38]. In CMT1C, LITAF is implicated in protein degradation [39]. LITAF also functions in cytokine production [40]. We speculate that because F16F9.1 is expressed in the EC, a tissue exposed to the environment, it could be involved in innate immune responses. Also, because *C. elegans *F16F9.1 is expressed in neurons, it is possible that the nematode could act as a model for CMT1C.

The other TF we identified is *hlh-8*, a helix-loop-helix TF related to human TWIST. TWIST was originally characterized in *Drosophila *as a gene involved in dorsal-ventral patterning [41]. TWIST TFs bind E-box *cis-*regulatory elements. In *C. elegans*, HLH-8 is important for regulating muscle, intestinal and anal muscle development. Consequently *hlh-8 *mutants exhibit defecation and egg-laying defects [42]. Several transcriptional targets of HLH-8 are known, including *cdh-4*, *egl-15*, C18B12.6, F08D12.7, *rbc-1*, *npr-10*, *dhs-5*, *sgcb-1*, *erv-46*, M60.6, *R02E4.1*, *rev-1*, and *myo-3 *[43]. Most of these genes are unlikely to be transcriptional targets of HLH-8 in the EC as they are not expressed in this cell. An exception is *cdh-4 *[14,15], which encodes a widely expressed cadherin, which is also expressed in the EC. Due to the limited number of HLH-8 targets in the EC, we can envisage a model where CEH-6 plays a role in directing the precise transcriptional outcomes of downstream TFs (*e.g. hlh-8*). In this role, CEH-6 could modulate target genes (*e.g. cdh-4*) specifically in a subset of ectodermal tissues including the EC.

Several of the genes that depend on their upstream octamer containing fragments for EC expression are also expressed in additional tissues. An interesting consequence of assaying the activity of truncated promoters is that loss of the octamer containing fragment sometimes led to loss of expression in multiple tissues including neurons as indicated in the cases of ZC395.10, *twk-36*, M176.5, and F16F9.1 (Additional file 4). This is not surprising as vertebrate orthologs of CEH-6 including Brn1 are involved in neuronal and kidney development [44,45].

Our strategy for studying the above genes involved comparing the expression patterns resulting from promoter truncations that either contain or remove the octamer. Analyzing promoter function by means of these truncations imposes some significant drawbacks; for example, we could not address the function of several candidate octamer elements because removing regions upstream of the octamer led to loss of EC expression. Also, because our promoter truncations deleted the octamer and some flanking regions, we cannot be certain that loss of EC-selective expression is the consequence of removing the octamer. However, because the 5' ends of the truncations were in general fairly close to the octamer, and because loss of EC-expression correlated with octamer deletion, we believe that these genes are likely dependent on octamers for their expression in the EC. To address the issue of whether these are indeed functional octamers, one could introduce point mutations within the octamer and assess the resulting consequences on EC-expression. However, we demonstrated in our mutagenesis experiments that certain point mutations are not sufficient to abolish EC expression in the context of the *aqp-8 *promoter. Another potential drawback from our approach is the fact that much of our study is based on expression patterns arising from transgenic *C. elegans *strains containing extrachromosomal arrays. Such arrays are susceptible to somatic transgene loss, which results in mosaic reporter expression. This mosaiscism has the potential to confound our analysis by under-representing the expression pattern. However, expression patterns resulting from genome-integrated transgenes (*aqp-8*^promote*r*^*::GFP*) are identical to the expression patterns in *aqp-8*^promote*r*^*::GFP *strains carrying extrachromosomal transgenes. Therefore, mosaic loss of the extrachromosomal transgene array is not likely an issue for analyzing changes in EC expression. Despite the potential shortcomings detailed above, our study revealed a set of genes whose expression likely depends on the octamer for expression in the EC; these genes are excellent CEH-6 candidate targets. In agreement with this notion, *twk-23*, one of the genes that we identified as dependent on an upstream octamer fragement, has been demonstrated, independently, to be regulated by CEH-6 [26].

Because our bioinformatic search did not bias the direction of the octamer, we discovered promoters in the forward and inverted orientation can be associated with EC-expression. Octamers in a forward orientation occur in the promoters of *aqp-8*, M176.5, *twk-36*, ZC395.10, *hlh-8*, C01B12.3, and C05D12.1, whereas inverted octamers are present in the promoters of F16F9.4 and R02F2.8. Although we could not determine from our small sample set whether the direction of the octamer has a functional consequence, there are possible implications related to direction of the octamer sequence. For example, octamers upstream of immunoglobulin light and heavy chain genes have directional preferences (ATGCAAAT and ATTTGCAT respectively) [46]. In addition, the human POU homeobox gene *Oct1 *is auto-regulated by two upstream octamers, which are also situated in inverted orientations. Although each site binds Oct1 with equal affinity, each of these sites has different effects on Oct1 expression [47].

It appears that transcriptional auto-regulation is a common mode of regulation among POU TFs [47-49]. Interestingly, we detected an octamer upstream of *ceh-6*'s ATG in *C. elegans*. Likewise, there is an octamer located in the regulatory region of the *C. briggsae *gene encoding a putative CEH-6 ortholog, providing evidence that auto-regulation might be conserved. However, these octamer are located within a predicted non-coding RNA gene (class RNAz) [50]. Nevertheless, it would be interesting to determine whether CEH-6, like other POU TFs is auto-regulated.

With our set of nine candidate octamers, we had the opportunity to determine whether residues flanking the element are conserved. Globally, the G/C content of *C. elegans *is 31% [51]. However, the regions flanking the octamers-associated with EC-expression contain a slightly higher G/C content (38%). Our alignments of these octamers revealed that despite the higher G/C content, some positions have preferences for A/T residues. Strikingly, directly 3' to the octamer, an A or T is always present. The conservation of this residue is consistent with the results of a previous study, which identified Oct1 binding preferences using a Systematic Evolution of Ligands by Exponential Environment (SELEX)-based *in vitro *binding approach [52]. Because of the enriched G/C content in octamer adjacent regions, it is likely that the observed preference for A/T at certain positions have relevance.

We tested the effects of targeted-octamer mutations on EC-expression. We found that several single-base substitutions did not affect the *cis-*regulatory element's ability to drive expression in the EC. Interestingly, we did not observe a change in expression level or pattern when position five of the octamer was mutated from a purine to purine (ATTT**G**CAT→ATTT**A**CAT). This variant of the octamer was demonstrated to be a binding site for the catfish class III POU TF, Oct2 [53]. Therefore, this residue change results in an octamer variant which retains the ability to interact with the POU_S _sub-domain binding consensus sequence (TG(C/A)ATattc) [54]. At the same residue position, a thymine replacement (ATTT**G**CAT→ATTT**T**CAT), led to weak GFP expression also restricted to the EC. This sequence was able to bind to Oct1 *in vitro *in an electrophoretic mobility shift assay (EMSA) [55]. However, in another study this variant of the octamer was not able to drive reporter expression in human cells [56]. These results, taken together, indicate that this motif variant is a sub-optimal POU TF binding site that can drive weak EC expression in *C. elegans*. A mutation at the sixth residue (ATTTG**C**AT→ATTTG**A**AT) also led to weak EC-localized expression [10]. This octamer variant is functional in the promoter of the *Drosophila *gene, Choline Acetyltransferase (ChAT). In the ChAT promoter, ATTTGAAT interacts with the POU homeobox TF, dPOU-19 [57]. All other single nucleotide substitutions at these two locations led to loss of GFP expression. However, a double residue replacement of these residues (ATTT**GC**AT→ATTT**AG**AT) could still drive expression, as indicated by weak GFP expression in the EC. There have been no previous reports of this dual nucleotide substitution variant associating with POU TFs and it is therefore a novel POU TF binding site variant. Overall, our mutagenesis assays indicate that the octamer *cis-*regulatory element could have a range of functional variants in *C. elegans*. Thus, identifying and characterizing promoters containing these octamer variants may reveal a larger group genes expressed in the EC.

Because variants of the octamer can influence EC-expression, we examined the *pgp-12 *promoter region in *C. elegans *more closely. Previously, it was demonstrated that *pgp-12 *expression is regulated by DCP-66/Ex-1 [8]. In this report, the TF/*cis-*regulatory element interaction was confirmed using *in vitro *and genetic approaches. Loss of either Ex-1 (located 238 bp upstream *pgp-12*'s ATG) or DCP-66 resulted in loss of EC expression [1]. We detected an octamer like sequence (ATTTCCAT) that partially overlaps the Ex-1 (-241 bp). We also identified this octamer-like sequence in the orthologous regions of *C. briggsae*. Using the Transcriptional Element Search System (TESS) [58] to identify predicted *cis-*regulatory elements, we found that the octamer-like sequence is indeed a potential target for octamer binding proteins. Additionally this sequence binds Oct1 *in vitro *[52]. In fact, Zhao *et al*. reported that promoter constructs encompassing Ex-1 at -241 bp results in strong reporter expression during all developmental stages [8]. They also studied the expression pattern resulting from a promoter region defined by a 5'-end 238 bp upstream of the ATG, thereby removing three nucleotides from the octamer-like sequence. Although this promoter still drove EC-expression, the intensity of the GFP reporter was greatly decreased in adult and larval worms. Additionally, embryonic expression was almost eliminated. Finally, a construct with a 5'-end 228 bp upstream of the ATG was not able to drive expression of GFP indicating the necessity of the Ex-1 (and the octamer-like sequence) for EC expression. Therefore, not only did this prior study define the role of the Ex-1 for EC-expression, but it also indirectly provided evidence that the octamer upstream of *pgp-12 *might affect EC-expression. This suggests that the DCP-66/Ex-1 and the octamer-directed transcriptional regulatory mechanisms co-operatively modulate the expression of *pgp-12 *in the EC. This model of concerted and redundant regulation of EC-expression may have relevance in other genes including, possibly, several genes within our phylogenetically defined candidate set.

## Conclusions

Overall, we determined that the octamer is likely responsible for the expression of several genes within the EC, an osmoregulatory organ analogous to the kidney. Because one of our candidate genes, *twk-36*, has been demonstrated to be a *bona fide *target of CEH-6 regulation, it would be interesting to determine whether CEH-6 is involved in the regulation of four other candidates. Although our candidates, for the most part, were chosen based upon perfect conservation of the octamer, we determined that several variants of the octamer can drive EC-expression. The existence of functional octamer variants indicates that future searches for octamer-driven genes should use a loosely defined octamer sequence. Overall, understanding conserved mechanisms of gene regulation that determine appropriate EC expression may provide insight into underlying transcriptional mechanisms that regulate transcription in analogous organs including the kidney.

## Methods

### Nematode strains and maintenance

*C. elegans *strains were maintained at 20°C on nematode growth media (NGM) plates inoculated with *E. coli OP50*. All manipulations were conducted using standard procedures [59]. For the list of *promoter::reporter *constructs used in this study, refer to Additional file 5.

### Generation of transgene constructs and strains

DNA constructs were generated using fusion PCR as previously described [60]. Promoter-containing sequences were fused upstream of the GFP-coding region in the *pPD95.67 *GFP-coding cassette. The *octamer::vit-2*^*promoter*^*::GFP *chimeric constructs were generated by PCR as follows. The forward PCR primers contain three tandem repeats of the octamer at the 5' end of a *vit-2*-promoterspecific sequence. The right primer of the *vit-2 *chimeric promoter constructs remained consistent between strains (vit2reverse -AGT CGA CCT GCA GGC ATG CAA GCT CGA CCT GAT GGC TGA ACC G). The chimeric promoters were fused to the GFP-coding region in the *pPD95.67*. The mutagenized octamer constructs were generated by substituting target nucleotides in the forward PCR primer.

All *C. elegans *microinjections were conducted on either an Olympus BH2-HLSH or a Zeiss 47 3016 invert microscope. The PCR constructs were injected into the syncitial region of the gonad. The final concentrations of the injection mix are 30 ng/μl of the target construct along with 100 ng/μl of the marker construct, *pCeh361 *(*dpy-5*(+)) [61], into the target strain *dpy-5*(*e907*) (CB907). Transgenic F_1_s (Dpy-5 rescued) were individually plated. Wild type F_2 _lines were selected to establish the transgenic lines. When available, we analyzed a second independently segregating transgenic line.

### Identification of all *C. elegans *genes with upstream octamers

All genes containing an octamer (ATTTGCAT or ATGCAAAT) within 1,200 bp upstream of a protein-coding gene in *C. elegans *were identified in WormBase, WS195.

### Identification of all genes with interspecies conserved upstream octamer

1,000 bp upstream of the ATG of all orthologous gene groups in the nematodes:*C. elegans, C. briggsae*, and *C. remanei *(WormBase WS195), were searched for the presence of octamers. A *C. elegans *promoter was considered if its *C. briggsae *and *C. remanei *counterparts both contain one or more upstream octamers. Sequences flanking the ATGs from these three *Caenorhabditis *species and the predicated motifs were loaded into a MySQL database using the GFF3 format http://www.sequenceontology.org/gff3.shtml. The comparative analysis was performed in programs written in Perl. We used the Bio::DB::GFF Perl module [62].

### Microscopy

All GFP-expression analyses were conducted on a Zeiss Axioscope equipped with a QImaging camera and the appropriate GFP optical filter sets. Worms were immobilized with 100 mM sodium azide (in water) immediately prior to imaging. All images were captured at 400× with identical camera and fluorescence settings for all images (exposure times are indicated in the Figures) using QCapture software. The GFP images from each transgenic strain are representative of their populations.

## Authors' contributions

AKM carried out the molecular genetic studies, conceived the study, and wrote the manuscript. DKT carried out the microinjections. RCJ helped to draft the manuscript. JSC performed the bioinformatics portion of the study. NC participated in the design of the study. DLB participated in the design and coordination of the study. All authors read and approved the final manuscript.

## Supplementary Material

Additional file 1 **5' deletion of regions containing upstream octamers**. Promoter regions that caused EC-expression were truncated in a 5'-manner. The constructs were either truncated in an octamer-targeted manner or in an unbiased manner. The 5' ends of the PCR primer and octamer locations are relative to the genes' ATGs. The stages of expression are designated as E: embryonic, L: larval, and A: adult. The expression intensity levels are designated as L: low, M: medium, or H: high. Click here for file

Additional file 2 **Genes with promoters containing conserved octamers**. Through comparisons of promoter regions between *Caenorhabditis *species, we found 165 genes in *C. elegans *that have conserved octamers. Click here for file

Additional file 3 **Expression patterns of genes containing interspecies conserved octamers in their upstream regions**. We analyzed the expression patterns of 107 genes that have upstream octamers. 64 promoters led to assayable levels of the GFP reporter. 25 of these had EC-expression with 13 expressing in the EC-exclusively. Click here for file

Additional file 4 **Testing of upstream octamers associated with EC expression**. We selected a subset of promoters to analyze from Additional file 2. These promoters were demonstrated to influence EC-expression. The third column represents the direction of the octamer sequence. Forward: ATTTGCAT, Reverse: ATGCAAAT. Upstream regions that drove EC expression were truncated from the 5' end in an octamer-targeted manner. The 5' end of the PCR primer and octamer location are relative to the genes' ATGs. The stages of expression are designated as E: embryonic, L: larval, and A: adult. The expression intensity levels are designated as L: low, M: medium, or H: high. Click here for file

Additional file 5 **Transgenic strains used in this study**. The transgenic strains used in this study are encompassed in this list. Click here for file
